# The Absence of Feces in Suckling Cats

**Published:** 1898-07

**Authors:** B. B. Bell


### THE ABSENCE OF FECES IN SUCKLING CATS.

Read before the Alumni Association of the Southern Medical College, July 22, 1898.

When a child, I was quite fond of cats, and like most young boys, was often so rough as to get myself scratched by the worried animals. Even in those days, I often wondered why one never perceived any odor or other sign of feces about a litter of young kittens. Yet in the case of half-grown cats, there are perhaps few boys who have not through mischief or roughness, provoked them to a discharge, the disagreeablelessness of which one is not likely to forget.

Last year a lady gave me a young cat, named Black Joe, a case of mistaken sex with the lady, no doubt. She was very affectionate and a fine mouser, so that I petted her a great deal. About two months ago, Joe gave birth to two kittens. She was in my bedroom, and when I carried her out, she mewed so plaintively to get back in, that I relented, thinking that I would seize such a favorable opportunity of finding out beyond a doubt what becomes of the little animal's feces.

I put the trio into a dry-goods box, in which I had previously placed some clean, white, lint-cotton. Now as I was at home every night during the first month, and the half of every day, and as my office and bedroom are within a few yards of each other, I had the little family under my eyes almost continually. At no time during those thirty days could I detect with either the sense of sight or that of smell, anything indicating either feces or urine. It has been suggested that the mother removed the discharges or licked them away. In this case, she stayed with her pets every night, and early next morning would take a stroll. I always watched her carefully and she carried nothing away. As for licking, we all know how cleanly cats are, from their standpoint, and how little likely the mother cat would be to swallow such abominations. Now, if she licked them away, without swallowing, where would they be? Moreover, I watched Joe often as she licked her offspring, she was very particular about keeping them clean, and was quite profuse in her caresses around the anus and genitals, but there was at no time any secretion to lick away.

When they were about a month old, one of the little ones died. He had never thrived and the intense June heat was too much for him. Just here is where I missed my opportunity! I should have examined the intestines for fecal matter. Unfortunately, the idea occurred to me only when it was too late. However, I continued to observe the other: He was soon able to get out of the box and crawl about the room: still no feces. Lest he might deceive me, since cats, in this matter, are secretive, I confined him to the room, while he and his mother spent the nights at my feet.

One day when he was about six weeks old, Joe, after the manner of cat mammas at this stage, brought in a lizard to her baby, but not all her arts could induce him to notice it.

The same afternoon, she brought him a rat. Instantly he became interested, but would not try to eat till she had exposed the flesh by biting its neck: then he sucked freely of the juice and blood. The next morning for the first time, I found a small, hard, reddish roll or cylinder of unmistakable cat-feces. The second day no tidbit was brought and the following day there were no feces. The third afternoon kitty received a young rabbit over which he had a high old time, growling and snarling, as kittens are wont to do. The next morning I had to remove more feces that were still more cat like. Well, to be brief, I have kept the little feline under almost constant surveillance, being under lock and key while I was away. I have likewise kept up with the mother; she has not brought in food every day, only two or three days out of the week, and I have seen feces at no time, save within twenty-four hours after such times.

I give these outlines in the hope that a more competent person may make a thorough investigation. I know it is deemed impossible for a highly organized animal to exist without throwing off solid excreta, but the history of science is only a succession of so-called impossibilities proven to be facts. The importance of the subject under discussion to our profession need not be enlarged upon.—B. B. BELL, M. D.

